# Cellulose Nanofibril-Based Biodegradable Polymers from Maize Husk: A Review of Extraction, Properties, and Applications

**DOI:** 10.3390/polym17141947

**Published:** 2025-07-16

**Authors:** Nthabiseng Motshabi, Gaofetoge Gobodiwang Lenetha, Moipone Alice Malimabe, Thandi Patricia Gumede

**Affiliations:** 1 Department of Life Sciences, Faculty of Health and Environmental Sciences, Central University of Technology, Bloemfontein 9301, South Africa; nthabiseng.motshabi.nm@gmail.com; 2 Division of Student Learning and Development, Faculty of Health Science, University of the Free State, Bloemfontein 9301, South Africa; setlharegg@ufs.ac.za; 3 Department of Chemistry, Faculty of Natural and Agricultural Sciences, University of the Free State, Qwaqwa Campus, Phuthaditjhaba 9866, South Africa; mokoenama@ufs.ac.za

**Keywords:** biodegradable polymers, polybutylene succinate, polylactic acid, cellulose nanofibrils (CNFs), maize husk

## Abstract

The environmental impact of petroleum-based plastics has driven a global shift toward sustainable alternatives like biodegradable polymers, including polylactic acid (PLA), polybutylene succinate (PBS), and polycaprolactone (PCL). Yet, these bioplastics often face limitations in mechanical and thermal properties, hindering broader use. Reinforcement with cellulose nanofibrils (CNFs) has shown promise, yet most research focuses on conventional sources like wood pulp and cotton, neglecting agricultural residues. This review addresses the potential of maize husk, a lignocellulosic waste abundant in South Africa, as a source of CNFs. It evaluates the literature on the structure, extraction, characterisation, and integration of maize husk-derived CNFs into biodegradable polymers. The review examines the chemical composition, extraction methods, and key physicochemical properties that affect performance when blended with PLA, PBS, or PCL. However, high lignin content and heterogeneity pose extraction and dispersion challenges. Optimised maize husk CNFs can enhance the mechanical strength, barrier properties, and thermal resistance of biopolymer systems. This review highlights potential applications in packaging, biomedical, and agricultural sectors, aligning with South African bioeconomic goals. It concludes by identifying research priorities for improving compatibility and processing at an industrial scale, paving the way for maize husk CNFs as effective, locally sourced reinforcements in green material innovation.

## 1. Introduction

The environmental harm caused by petroleum-derived plastics has become one of the most pressing global sustainability challenges [1]. These conventional plastics are versatile and inexpensive. However, they persist in the environment for hundreds of years, contributing to land and marine pollution, threatening biodiversity, and disrupting ecosystems [2]. Moreover, their production is heavily reliant on fossil fuels, contributing to greenhouse gas emissions and climate change [3]. In response to these issues, the search for sustainable, degradable alternatives to conventional plastics has gained significant attention across research, industry, and policy domains [4].

Biodegradable polymers have emerged as promising alternatives due to their ability to decompose under natural environmental conditions [5]. Materials such as polylactic acid (PLA), polybutylene succinate (PBS), and polycaprolactone (PCL) are among the most studied biopolymers. They offer viable functionality in packaging, agricultural films, and biomedical applications [6,7]. However, despite their environmental advantages, many of these biopolymers exhibit limitations such as low mechanical strength, poor thermal resistance, and high production costs, which hinder broader industrial adoption [8].

To overcome these challenges, reinforcement of biodegradable polymers with natural fibres, especially nanocellulose, has been widely explored. Cellulose is the most abundant biopolymer on earth. It is biodegradable, biocompatible, and available from a diverse range of biological sources, including wood, agricultural residues, algae, and bacteria [9]. Among the various nanoscale forms of cellulose, cellulose nanofibrils (CNFs) have received considerable attention due to their high aspect ratio, excellent mechanical properties, and large surface area. When incorporated into polymer matrices, CNFs can significantly enhance tensile strength, thermal stability, and barrier properties, while maintaining biodegradability [10].

The source of cellulose plays an important role in determining the properties, cost-effectiveness, and sustainability of the resulting CNFs. While wood pulp remains a conventional source, growing attention is being directed toward agricultural waste materials as renewable and low-cost feedstocks. Residues such as rice husk, wheat straw, and sugarcane bagasse have already been investigated for CNF extraction, with promising results in terms of reinforcing capability and environmental impact reduction [11].

In this context, maize husk, which is a lignocellulosic byproduct largely available in agricultural economies such as South Africa, presents a compelling opportunity. Maize is one of the most cultivated crops in the country, and its processing generates substantial husk waste that is often discarded, incinerated, or underutilised for low-value applications. It is rich in cellulose and hemicellulose and offers a renewable, underexploited resource for producing CNFs that could be valorised into high-performance, biodegradable composite materials [12]. Reclaiming this waste material aligns with global environmental goals and also supports local economic development, especially in rural farming communities.

Despite its potential, research on CNFs derived from maize husk remains limited, especially within the South African context. There is a need for systematic studies that examine the extraction methods, material characteristics, and performance of maize husk CNFs in polymer matrices. Moreover, aligning such studies with local waste valorisation strategies and green manufacturing practices could offer both scientific and socio-economic impact [13].

This review aims to bridge that knowledge gap by providing a comprehensive analysis of current research on maize husk-derived nanocellulose and its integration into biodegradable polymer systems. It examines extraction techniques, structural and mechanical properties, composite processing strategies, and potential applications, with a focus on local relevance and environmental sustainability.

By highlighting the untapped potential of maize husk as a feedstock for advanced biocomposites, this review contributes to the broader discourse on the circular bioeconomy and materials innovation in the Global South. Furthermore, this review includes numerical data on key composite performance indicators such as mechanical properties, crystallinity, and biodegradation behaviour compiled from the literature and presented in graphical formats to enhance clarity and facilitate comparative analysis.

To achieve this, the review is structured as follows: Section 1 provides an introduction that outlines the motivation, scope, and significance of using cellulose nanofibrils (CNFs) derived from agricultural residues, especially maize husk, for the development of sustainable materials. Section 2 focuses on maize husk as a renewable and abundant source of cellulose, highlighting its chemical composition and potential as an underused lignocellulosic biomass. Section 3 reviews various extraction methods for isolating CNFs from various lignocellulosic biomasses, including mechanical, chemical, and enzymatic techniques, with an emphasis on efficiency, scalability, and environmental impact. Subsequently, Section 4 summarises the physicochemical, morphological, thermal, and mechanical properties of maize husk-derived CNFs reported in the literature. Section 5 addresses the processing methods and challenges associated with incorporating these CNFs into biodegradable polymer composites. Section 6 focuses on the role of CNFs as reinforcements in biodegradable polymers, discussing their structure, properties, and reinforcing mechanisms compared to traditional fillers. Section 7 explores the applications of CNF-based biocomposites within the South African context, emphasising areas such as packaging, agriculture, and environmental remediation. Finally, Section 8 concludes with a synthesis of the key findings and offers future perspectives, identifying current gaps in research and proposing directions for further development and policy support in this emerging field.

## 2. Maize Husk as a Renewable Source of Cellulose

Agricultural residues are increasingly recognised as valuable lignocellulosic feedstocks for sustainable material production due to their abundance, renewability, and biodegradability [14,15]. Among these, maize husk, which is an underused by-product of maize processing, has gained attention for its potential as a cellulose-rich substrate in biopolymer composite applications. While the structural composition of maize husk shares similarities with other lignocellulosic biomass, it presents distinct advantages in terms of cellulose accessibility, fibre morphology, and ease of processing [16]. As shown in Table 1, the cellulose content of maize husk is approximately 35%, placing it in a comparable range to commonly studied agricultural residues such as wheat straw (36%), rice straw (39%), and corn stover (33%) [15]. However, maize husk exhibits a higher hemicellulose content (36%) and a relatively low lignin concentration (12%), which may facilitate more efficient delignification and fibre extraction processes. This composition suggests that maize husk offers a favourable balance for the production of cellulose nanofibril (CNF)—sufficient cellulose to yield reinforcing fibres and reduced lignin levels that facilitate more efficient delignification and fibre extraction [17]. The higher hemicellulose content also enhances the reactivity and flexibility of the biomass during chemical or enzymatic pretreatment, reducing both environmental impact and operational costs [18].

The valorisation of maize husk aligns well with circular economy principles and waste-to-resource strategies, especially in maize-producing regions such as sub-Saharan Africa, where this biomass is often discarded or incinerated, contributing to environmental degradation [20,21]. Its local availability, favourable chemical composition, and suitability for low-energy processing render maize husk a compelling and sustainable candidate for CNF production and subsequent use in biodegradable polymer reinforcement.

## 3. Extraction Methods for CNF from Lignocellulosic Biomasses

The extraction of cellulose nanofibrils (CNFs) from lignocellulosic biomass involves a range of mechanical, chemical, and physicochemical methods. Each method is tailored to the structural complexity and chemical profile of the source material [22]. In particular, CNFs are isolated through high-pressure homogenisation, grinding, or enzymatic pretreatment, while cellulose nanocrystals (CNCs), also derived from the same biomass, are generally produced via acid hydrolysis. In the case of maize residues such as husk, stalk, tassel, and corncobs, several extraction strategies have been evaluated, reflecting increasing interest in valorising this agricultural waste stream into high-value nanomaterials [14,23].

Table 2 summarises key extraction methods used across various maize biomass types in South Africa, detailing the techniques used, nanocellulose properties obtained, and their performance outcomes in composite systems. The table provides a comparative view of extraction routes, such as acid hydrolysis, alkali treatment, enzymatic delignification, and mechanical fibrillation, along with their associated crystallinity index, CNF yield, and thermal stability. Corncob and husk residues demonstrate high crystallinity and reinforcement potential, making them strong candidates for polymer applications.

The choice of extraction route significantly impacts the morphology, crystallinity, and reinforcement potential of the resulting nanocellulose. For instance, acid hydrolysis of corncobs has yielded CNCs with crystallinity as high as 79.11% [24], while alkali treatment of tassel fibres increased cellulose content from 41% to 56% [25]. Moreover, the incorporation of CNCs derived from corn husk improved the Young’s modulus of natural rubber composites by over 100% [26], demonstrating their potential as effective reinforcement agents in biodegradable polymer systems. In South Africa, the decentralised availability of maize biomass offers a strategic advantage, but technological and economic barriers remain. Scaling these extraction processes for local production will require low-energy, water-efficient methods and policy alignment with national circular economy targets.

The diversity of maize biomass used, ranging from tassel and stalk to husk and corn-cob, highlights its versatility as a lignocellulosic source. However, a persistent limitation is the absence of standardised benchmarks for assessing yield, crystallinity, environmental impact, and scalability. This shows the urgent need for harmonised methodologies, greener extraction technologies, and application-driven optimisation, especially for biodegradable polymer composites aligned with circular bioeconomy goals [27,28].

**Table 2:** The extraction of cellulose from maize residues (tassel, stalk, and stem).

Extraction: Source and Cellulose	Method	Results	References
Cellulose fibres from the maize tassel	Alkali	Cellulose content increased from 41% to 56%, following alkali treatment.	[25]
Cellulose nano-whiskers (CNWs) from maize stalk	Chemical acid hydrolysis	CNWs have diameters between 3 and 7 nm, length between 150 and 450 nm.	[29]
CNCs from corn (*Zea mays*)	Alkali-treated, bleached, acid hydrolysis (sulfuric acid)	Increased Young’s modulus of natural rubber composites from 0.89 ± 0.15 MPa to 1.98 ± 0.73 MPa with the addition of 2 wt% CNCs. CNCs length of 940 ± 70 nm, width of 6 ± 2 nm, high aspect ratio of 157.	[26]
CNCs from corncobs	Chemical acid hydrolysis	Higher crystallinity (79%), Optimum yield of 81% at a temperature of 30 °C, 30.13 min reaction time, and 46 wt% sulfuric acid concentration.	[24]
CNCs from corncobs	Liquid hot water, Alkali treatment (temperature (150–200 °C), time (10–60 min), (3–10% *w*/*w*) NaOH (2 wt%) at 90 °C for 90 min	Yield of 56% at 200 °C, 10% *w*/*w*, and 60 min, surface morphology showed a more porous and rougher surface, and the crystallinity index of 57%.	[28]
α-cellulose from maize (*Zea mays* L.) husk	Chemical acid hydrolysis	98% α-cellulose, 0.19% β-cellulose, and 1.86% γ-cellulose, 41% carbon, 3% hydrogen, 0.7% nitrogen, 0.07% sulphur, and 55.28% oxygen.	[27]

## 4. Properties of Maize Husk-Derived CNF

The physicochemical properties of cellulose nanofibrils (CNFs) are important for their functionality in polymer nanocomposites. They influence interfacial bonding, mechanical reinforcement, and biodegradation behaviour. CNFs derived from agricultural residues exhibit diverse structural and morphological characteristics based on source material and extraction method. As shown in Table 3, maize husk-derived CNFs have properties that are competitive with, and in some cases comparable to, those obtained from other lignocellulosic materials such as corncob, sugarcane bagasse, and apple pomace.

CNFs extracted from maize husk through sequential alkali treatment, bleaching, and sulfuric acid hydrolysis have been reported to possess high aspect ratios, with lengths of approximately 940 ± 70 nm [26]. While the crystallinity index was not reported in that specific study, comparable sources, such as corncob, exhibited high crystallinity values of 79.11% [24], suggesting that similar treatment protocols applied to maize husk could yield CNFs with excellent crystalline order. High crystallinity in CNFs is directly associated with improved mechanical reinforcement potential when incorporated into polymer matrices, as it enhances stiffness, tensile strength, and dimensional stability [30,31].

Across other biomass types, variation in crystallinity (ranging from ~47% to over 80%) and particle dimensions shows the sensitivity of CNF properties to processing parameters. For instance, cellulose nano-whiskers extracted from maize stalks using acid hydrolysis were found to be significantly smaller in diameter (3–7 nm) and length (150–450 nm), suggesting that even within the maize plant, anatomical part and method used can yield markedly different nanostructures [29]. Additionally, the morphological characteristics of CNFs, such as needle-like, rod-like, or flake-like structures, are shaped by the interplay between acid concentration, reaction temperature, sonication intensity, and pre-treatment duration [32,33].

Maize husk CNFs stand out for their promising length-to-diameter ratio, which is advantageous for percolation in polymeric matrices, resulting in enhanced load transfer and barrier properties. However, more studies are needed to comprehensively characterise their crystallinity, thermal stability, and surface chemistry. Notably absent from the current literature are systematic investigations into the influence of husk-derived CNFs’ surface charge (e.g., sulphate groups from acid hydrolysis) on matrix compatibility, especially in hydrophobic biodegradable polymers.

The variability in properties across studies, even when using similar sources, highlights the need for standardised protocols for CNF extraction and characterisation. This would enable more meaningful comparisons across the literature and inform tailored application development. Furthermore, studies directly linking the structural properties of maize husk CNFs with performance metrics in nanocomposite systems remain limited, representing a significant gap in the field.

In summary, while maize husk-derived CNFs demonstrate favourable properties that position them as strong candidates for reinforcing biodegradable polymers, further research is required to fully map the processing–structure–performance relationships. Establishing these links will be essential to optimising their use in sustainable materials engineering and enhancing their competitiveness with more widely studied nano-cellulose sources.

**Table 3:** Properties of cellulose.

Cellulose	Source	Method of Extraction	Crystallinity (%)	Diameter (μm)	Length (nm)	Morphology	Reference
CNC	Maize husk	Alkali-treated, bleached, and hydrolysed to CNCs using sulfuric acid.			940 ± 70		[26]
CNC	Apple pomace	Alkaline NaOH (6–12%), (30–90 °C), (30–240 min) Response surface methodology (RSM) Acid hydrolysis Ultrasonication treatments	78	0.0079	28 ± 2.03	needle-like structure	[32]
CNC	Corncob	Acid hydrolysis Delignification (alkali and bleaching pretreatment)	79			needle-like	[24]
Cellulose acetate	Cajuput twigs, sugarcane bagasse	Pre-hydrolysis (NaOH) Pulping and elemental chlorine-free bleaching, iodine (I) as a catalyst	76	30			[34]
Cellulose nano-whiskers Cellulose nano-whiskers	Maize	Cutting mill (mechanical) Chemical extraction Bleaching		0.007	50–450		[29]
Cellulose	Sugarcane bagasse	Alkaline curing, (0.5, 1.5, 2.75, and 4%) NaOH, 120 °C, (15, 30, and 45 min) Optimum conditions: 2.75% NaOH, 120 °C, 45 min	32	13			[35]

## 5. Processing of CNF-Reinforced Biodegradable Polymer Composites

The incorporation of cellulose nanofibrils (CNFs) into biodegradable polymer matrices such as polylactic acid (PLA), polybutylene succinate (PBS), and polycaprolactone (PCL) is a well-established strategy for enhancing the mechanical, thermal, and barrier properties of bio-based plastics [23,36]. However, the efficiency of reinforcement is largely dependent on the dispersion of CNFs within the polymer and the quality of interfacial adhesion. The hydrophilic nature of CNFs and the hydrophobic character of most biodegradable polyesters present a fundamental compatibility challenge, often leading to agglomeration and limited stress transfer. CNFs derived from maize husk offer unique potential due to their nanoscale dimensions, high crystallinity, and modifiable surface chemistry [37]. Nevertheless, their effectiveness depends on surface modifications and processing techniques that can overcome interfacial incompatibility. There is a growing need to develop maize-husk-specific composite protocols that consider CNF purity, surface functionality, and thermal sensitivity during processing.

### 5.1. Polymer Selection and Matrix–Filler Interactions

Polylactic acid has received extensive attention in the context of CNF reinforcement due to its mechanical strength, biodegradability, and commercial availability. However, it is brittle and thermally unstable. Several studies [38] report that the incorporation of CNFs at low loadings (1–5 wt%) enhances the Young’s modulus and tensile strength of PLA. This enhancement is attributed to the rigid nature of CNFs and their ability to restrict polymer chain mobility. However, this restriction also leads to reduced elongation at break [39], which is a recurring challenge in such systems.

In PBS and PCL matrices, CNFs also act as nucleating agents, promoting crystalline phase formation that improves stiffness and barrier performance [40]. While trends are generally consistent across polymers, the magnitude of reinforcement depends strongly on matrix-filler compatibility and dispersion quality. A notable limitation across all matrices is the intrinsic incompatibility of untreated CNFs with hydrophobic polymers, leading to phase separation, agglomeration, and ineffective stress transfer [11]. This reinforces the need to tailor filler-matrix compatibility strategies based on polymer chemistry, CNF origin, and target application. Despite advancements, there is still a lack of consensus on optimal compatibilisation strategies for agricultural waste-derived CNFs such as those from maize husk, highlighting a critical research gap.

### 5.2. Surface Modification Strategies

To address compatibility issues, researchers have proposed various chemical modifications, including acetylation, silanisation, esterification, and grafting of CNFs with oligomers such as PLA and PCL. These treatments aim to reduce the hydrophilicity of CNFs and improve their dispersion in hydrophobic matrices [41]. PLA-grafted CNFs, for instance, have been shown to improve interfacial adhesion and increase composite tensile strength by up to 30% compared to unmodified CNFs [42]. TEMPO-mediated oxidation introduces carboxyl groups, enhancing hydrogen bonding potential and improving dispersion in matrices like PBS [43]. This method offers a greener alternative to more aggressive chemical modifications, though its industrial scalability remains limited.

With maize husk-derived CNFs, the role of surface chemistry becomes even more pronounced. The authors of [44] observed that alkaline-bleached CNFs had improved miscibility with PBS due to increased surface hydroxyl group exposure. Conversely, untreated maize husk fibres led to phase separation and early tensile failure. This suggests that maize husk CNFs require not just fibrillation but also deliberate chemical tailoring to unlock their reinforcing potential. However, few studies have directly compared the performance of different surface treatments (e.g., silanisation vs. esterification vs. TEMPO oxidation) on maize husk CNFs, indicating a critical opportunity for optimisation and standardisation.

### 5.3. Processing Techniques and Dispersion Quality

The method of composite fabrication plays a crucial role in determining CNF dispersion and overall material properties. Solution casting, melt compounding, and electrospinning are commonly used. Solution casting provides excellent dispersion control, especially in hydrophilic polymer systems, and is frequently used for laboratory-scale investigations [12]. However, its limited scalability and solvent usage are barriers to commercial implementation. Melt compounding, by contrast, is industrially relevant and scalable, but it poses challenges related to CNF thermal degradation and aggregation under high shear and temperature [45]. Fine-tuning processing parameters such as residence time, screw configuration, and compatibilizer concentration is essential for preserving CNF integrity and achieving uniform dispersion. Electrospinning has gained traction for biomedical and tissue engineering applications, where nanostructured scaffolds are desirable [46]. Its ability to align CNFs within fibres provides anisotropic reinforcement, but poor bulk dispersion and low throughput limit broader applicability.

**Figure 1:**
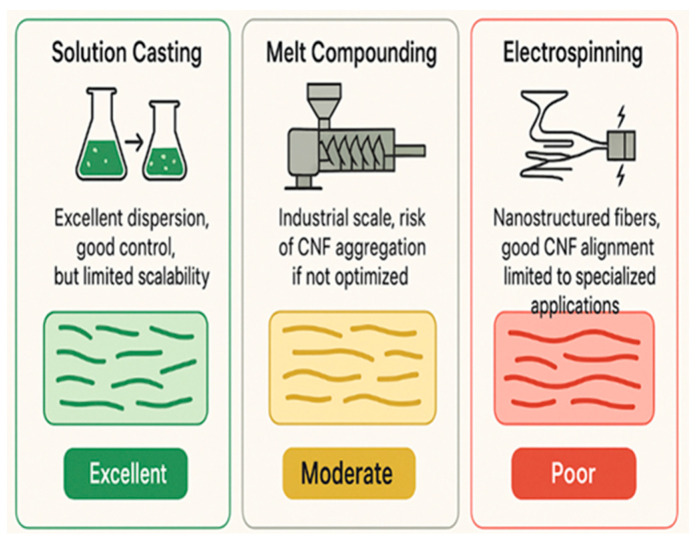
Comparative assessment of common processing techniques used for CNF-reinforced biodegradable composites [image generated by the author using ChatGPT (OpenAI, 2025)].

Figure 1 presents a comparative assessment of the three major processing methods such as solution casting, melting compounding, and electrospinning. Melt blending involves heating materials above their melting or glass transition temperatures and mixing them to achieve a homogeneous dispersion, making it cost-effective and environmentally friendly [45]. Whilst electrospinning uses high voltage to create charged jets of polymer solutions that form fibres, collected into non-woven mats [47]. In the case of solution casting, it disperses CNFs in a solvent and uses external forces like shear or electric fields to align them, enhancing mechanical strength and utilises solvent evaporation [48].

Maize husk-derived CNFs add complexity due to variable composition and thermal stability. Residual hemicellulose, for example, can promote thermal degradation during extrusion. Pre-drying, solvent exchange, and use of compatibilisers such as maleic anhydride-grafted PLA have been effective in improving dispersion and maintaining mechanical properties [49]. However, comprehensive processing guidelines tailored to maize husk CNFs are still lacking in the literature, pointing to a need for deeper exploration of formulation–process–property relationships.

### 5.4. Composite Property Enhancements

When dispersion and interfacial adhesion are well-managed, CNFs contribute significantly to mechanical strength, stiffness, and barrier performance. In PLA/CNF systems, CNFs act as nucleation sites that increase crystallisation rates and improve thermal resistance [50]. Crystallisation temperature and degree of crystallinity may increase by as much as 20% at CNF loadings below 5 wt%. In PBS composites containing maize husk CNFs, tensile modulus improvements of up to 40% and reductions in water vapour transmission rate by 30% have been reported [51]. These improvements are strongly correlated with the degree of CNF fibrillation and dispersion uniformity. However, CNF loadings above 5 wt% often result in agglomeration and embrittlement due to the formation of stress-concentrating domains. This non-linear relationship between CNF loading and property enhancement shows the importance of optimisation. Future studies should systematically map mechanical and barrier performance against dispersion metrics to inform formulation guidelines.

### 5.5. Biodegradability and Environmental Impact

CNFs are not only reinforcing agents but also biodegradable entities that promote hydrolytic degradation of polymer matrices. Their hydrophilic nature increases water uptake and matrix porosity, accelerating hydrolysis and facilitating microbial access [52]. This dual role of CNFs, as performance enhancers and biodegradation facilitators, makes them valuable in environmentally sensitive applications. Importantly, CNFs do not introduce persistent microplastic residues, distinguishing them from inorganic or synthetic fibre reinforcements. Their presence can enhance disintegration under composting or soil burial conditions, aligning with circular economy principles.

**Figure 2:**
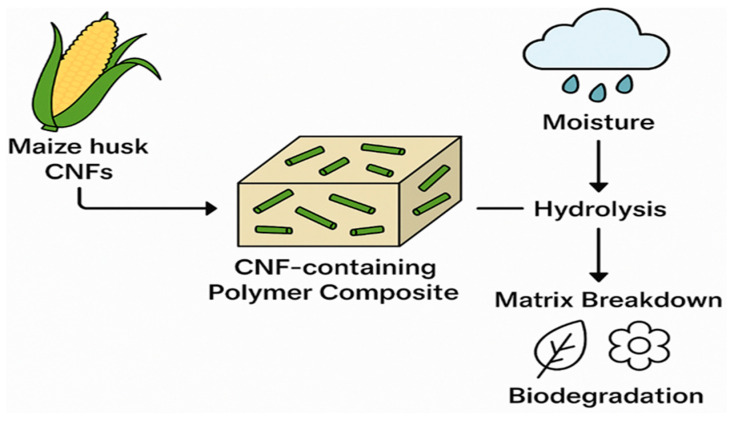
Schematic representation of the biodegradation pathway of CNF-containing biodegradable polymer composites [image generated by the author using ChatGPT (OpenAI, 2025)].

Figure 2 illustrates the biodegradation mechanism of CNF-reinforced composites: (i) moisture penetration into the polymer matrix, (ii) hydrolysis of polymer chains facilitated by CNF-induced porosity, (iii) microbial colonisation and enzymatic activity, and (iv) final mineralisation into CO_2_, water, and biomass. This figure clarifies the role of CNFs in initiating and sustaining the degradation process. Despite these advantages, few studies report long-term environmental behaviour of composites under varied disposal scenarios (e.g., marine environments, anaerobic digestion), suggesting an urgent need for broader biodegradation assessments that include maize husk-derived CNF composites.

In summary, the literature shows that CNFs, especially those sourced from maize husk, can significantly improve the performance of biodegradable polymers, provided that interfacial compatibility and dispersion are adequately managed. Future research should focus on refining green surface modification techniques and exploring synergistic interactions in ternary blends (e.g., PLA/PBS/CNF systems), with special attention to scalability and processing feasibility.

## 6. Cellulose Nanofibrils (CNFs) as Reinforcements for Biodegradable Polymers

**Figure 3:**
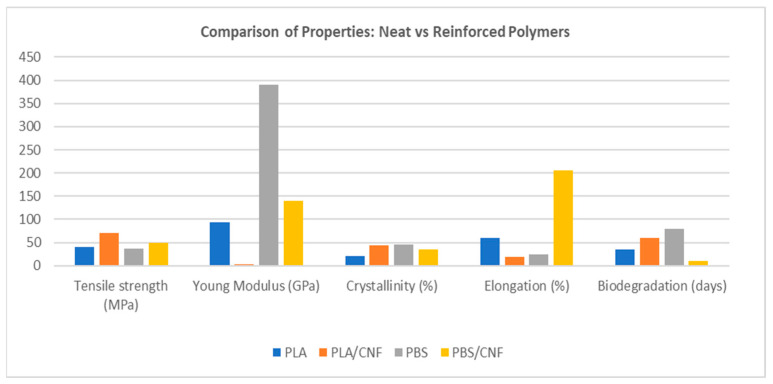
Comparison of properties of neat PLA, neat PBS, and reinforced biodegradable polymers. (**PLA**: Tensile strength: 40 MPa [61]; Young’s modulus: 94 GPa [38]; crystallinity: 21% [62]; elongation: 60% [38]; biodegradation: 35 days [63]. **PLA/CNF**: tensile strength: 71 MPa [64]; Young’s modulus: 4 GPa [64]; crystallinity: 44% [65]; elongation: 20% [66]; biodegradation: 60 days [67]. **PBS**: tensile strength: 37 MPa [68]; Young’s modulus: 390 GPa [69]; crystallinity: 45% [70]; elongation: 25% [71]; biodegradation: 80 days [72]. **PBS/CNF**: tensile strength: 60 MPa [23]; Young’s modulus: 140 GPa [23]; crystallinity: 36% [73]; elongation: 205% [74]; biodegradation: 10 days [75].).

The incorporation of cellulose nanofibrils (CNFs) into biodegradable polymer matrices has emerged as an effective strategy to enhance the performance of bioplastics such as polylactic acid (PLA), polybutylene succinate (PBS), and polycaprolactone (PCL). CNFs possess distinctive characteristics, such as a high aspect ratio, excellent tensile strength, low density, and reactive surface chemistry, which collectively enable them to function as highly efficient reinforcing agents in polymer nanocomposites [53,54]. Numerous studies have demonstrated that CNFs significantly improve the mechanical, thermal, and barrier properties of biodegradable polymers, thereby addressing some of the inherent performance limitations associated with neat bioplastics [55,56]. The reinforcing capability of CNFs is primarily attributed to their nanoscale dimensions and highly crystalline domains, which facilitate efficient stress transfer across the matrix–filler interface when adequate dispersion and strong interfacial adhesion are achieved [36]. For example, [26] reported that low CNF loading levels, typically between 1 and 5 wt%, can lead to improvements in tensile strength, Young’s modulus, and thermal stability in PLA and PBS matrices. These improvements include increases of up to 80% in tensile strength, 60% in Young’s modulus, depending on the polymer system and CNF loading. Figure 3 shows a comparative summary of key performance indicators such as tensile strength, modulus, crystallinity, elongation, and biodegradation for PLA and PBS before and post CNF reinforcement. The data highlights the improvements conferred by CNFs, with PBS/CNF composites showing superior elongation and biodegradation resistance. Such enhancements are advantageous in applications requiring durability and thermal resistance, including packaging and agricultural materials [57]. Moreover, CNFs can act as nucleating agents, promoting crystallisation in semi-crystalline bioplastics and consequently improving dimensional stability and processability [50].

Despite these benefits, the hydrophilic nature of CNFs and the hydrophobicity of most biodegradable polyesters often result in poor dispersion and weak interfacial bonding, limiting composite performance. To overcome this challenge, various surface modification techniques have been developed, including acetylation, silanisation, and grafting with compatibilisers, which enhance compatibility with polymer matrices [58]. For instance, TEMPO-mediated oxidation introduces carboxylate groups on the CNF surface, improving dispersibility in polar polymer matrices through hydrogen bonding interactions [59]. Alternatively, esterification with aliphatic or aromatic acids has been shown to increase CNF hydrophobicity, thereby enhancing interfacial adhesion in matrices such as PLA and PCL [37].

Processing methods also play an important role in determining the ultimate properties of CNF-reinforced composites. Techniques such as solution casting, melt compounding, and electrospinning have been widely reported, each presenting distinct advantages and limitations. While solution casting often facilitates superior initial dispersion of CNFs, melt compounding is preferred for industrial scalability and commercial applications. Electrospinning allows for the alignment of CNFs within nano-fibre mats, producing anisotropic mechanical properties desirable for specialised bio-medical scaffolds [60].

Recently, attention has shifted towards CNFs derived from non-traditional sources, including agricultural residues such as maize husk, due to their low cost, renewability, and local availability. Although most research has focused on wood- or cotton-derived CNFs, emerging studies suggest that agro-waste-derived CNFs can achieve comparable or even superior reinforcement performance when properly extracted and integrated into biopolymer matrices. For example, CNFs exhibit a high crystallinity index and aspect ratio, which translates to enhanced stress transfer and thermal resistance in PLA and PBS composites [23,36]. This approach aligns with Sustainable Development Goals by valorising agricultural waste streams and reducing dependence on conventional raw materials.

## 7. Applications of CNF-Based Biocomposites in the South African Context

The use of cellulose nanofibril (CNF)-based biodegradable composites in the South African context aligns with national imperatives around environmental sustainability, circular economy models, and local beneficiation of agricultural waste. CNFs derived from maize husk, which is a widely available agro-waste stream in South Africa, present a compelling value proposition for replacing petroleum-based plastics in packaging, agriculture, healthcare, and sanitation sectors. The literature shows several key application domains for CNF-reinforced biodegradable polymers, but contextualising these within South Africa’s developmental priorities reveals additional layers of relevance and opportunity.

### 7.1. Packaging Sector and Plastic Waste Management

Globally, the packaging sector is one of the largest consumers of single-use plastics, a pattern mirrored in South Africa, where plastic packaging constitutes over 60% of post-consumer plastic waste [76]. Biodegradable polymers such as PLA and PBS, reinforced with CNFs, have demonstrated significant promise in packaging applications due to their improved tensile properties, transparency, and barrier resistance [77]. Studies show that CNF additions can reduce oxygen and moisture permeability by 20–60%, depending on polymer type and nanofibril dispersion quality [58]. These enhancements make CNF-based composites viable candidates for food packaging, where shelf life and structural integrity are critical.

In the South African context, integrating CNFs from maize husks into packaging materials offers a dual advantage: it diverts agricultural waste from landfills and reduces dependence on imported bio-fillers. Furthermore, policy frameworks such as the Extended Producer Responsibility (EPR) regulations under the National Environmental Management: Waste Act (Act 59 of 2008) [78] incentivise producers to adopt sustainable packaging solutions, making CNF composites an attractive option for compliance and environmental stewardship.

### 7.2. Agricultural Films and Biodegradable Mulch

Biodegradable polymers have been extensively explored for agricultural films, including mulch and greenhouse covers, where controlled degradation and improved soil health are desired. CNF-reinforced PLA/PBS blends have demonstrated enhanced mechanical durability under UV exposure and microbial degradation in soil [79]. This is relevant to smallholder and commercial farming operations in South Africa, where plastic pollution from polyethene mulch films has emerged as a growing concern. Given the role of maize as a staple crop in South Africa, the valorisation of maize husk into CNF for agricultural film reinforcement represents a closed-loop innovation. The local production of CNF mulch films could significantly reduce imports and stimulate rural bio-economies, especially in provinces such as Mpumalanga, North West, and Free State, where maize farming is prevalent.

### 7.3. Biomedical and Health Applications

Biodegradable polymers reinforced with CNFs are increasingly studied for biomedical applications such as wound dressings, drug delivery systems, and tissue scaffolds, owing to CNFs’ biocompatibility, moisture retention, and structural properties [60,80,81]. In electrospun or film-based composites, maize husk-derived CNFs can serve as low-cost reinforcing agents that do not compromise the sterility of the material or degradation profile.

South Africa’s dual burden of disease and resource limitations in public health infrastructure provide fertile ground for low-cost, sustainable biomaterials. Locally produced CNF composites could be harnessed for single-use sanitary products or wound dressings in primary healthcare, especially in under-resourced clinics and mobile health units. However, this would require stringent validation for cytotoxicity, sterility, and regulatory approval, which remain underdeveloped in the current literature.

### 7.4. Public Sector Procurement and Green Innovation

The South African government has prioritised green procurement through its Green Economy Accord and Bio-Economy Strategy, which promote the use of renewable materials and support research-industry linkages. CNF-based bioplastics are well-positioned for public procurement in packaging, sanitation, and service delivery contracts, especially where biodegradability and local manufacturing are desired. As highlighted by [82], localised bio-innovation must be embedded within industrial policy to support technology adoption. Maize husk-derived CNFs, produced through scalable and low-cost methods, offer a compelling route for green public sector procurement. Municipal waste programmes, national school feeding schemes, and agricultural extension programmes can all benefit from biodegradable packaging, food trays, and planting aids made from PLA/CNF or PBS/CNF blends, contributing to job creation and environmental protection simultaneously.

### 7.5. Challenges and Future Opportunities

Despite these opportunities, several challenges remain. South Africa’s biopolymer sector is still nascent, with limited local PLA or PBS production capacity. Most studies rely on imported biopolymers, which elevate costs and reduce feasibility for large-scale roll-out. There is also a need for policy alignment between agriculture, environment, and industrial development departments to facilitate biomass valorisation and technology transfer. Moreover, public awareness around biodegradable materials remain low, necessitating community engagement and behavioural change interventions. Future opportunities include the establishment of decentralised CNF processing units in maize-producing regions, integration of CNF biocomposites in green building materials, and the expansion of academic–industry partnerships focused on end-use testing, life cycle analysis, and circular economy modelling. Multilingual public engagement campaigns could help bridge the knowledge–practice gap in communities most affected by plastic waste and agricultural underutilization.

## 8. Conclusions

The valorisation of maize husk into cellulose nanofibrils (CNFs) and their incorporation into biodegradable polymer matrices represents a critical nexus between waste management, materials innovation, and sustainable development. A growing body of literature shows that CNFs, due to their exceptional mechanical properties, high aspect ratio, crystallinity, can significantly enhance the thermal, mechanical, and barrier performance of biodegradable polymers such as polylactic acid (PLA), polybutylene succinate (PBS), and polycaprolactone (PCL).

These enhancements position CNF-based nanocomposites as viable alternatives to conventional, petroleum-based plastics in a range of sectors, such as packaging, agriculture, biomedical devices, and green infrastructure.

Within the context of maize husk, the review has shown that CNFs extracted from maize husk using chemical, mechanical, or chemo-mechanical methods offer competitive performance characteristics, comparable to CNFs derived from traditional lignocellulosic sources such as wood pulp or cotton. Their use in PLA/PCL/PBS blend matrices improves composite performance and also supports bioeconomy goals, local beneficiation of agro-waste, and the circular economy imperatives laid out in South Africa’s national development and environmental strategies.

This review has addressed a critical knowledge gap by providing a comparative synthesis of CNF extraction techniques, structural and functional properties, composite processing methods, and application relevance, supported by quantitative data visualisations (e.g., crystallinity, tensile strength, and biodegradation trends). The inclusion of data from maize husk-based CNFs and their contextualisation within South African developmental needs distinguishes this work from more generalised reviews.

Despite this promise, several gaps remain in both the literature and the translational landscape. Firstly, while numerous studies have established the physicochemical and mechanical superiority of CNF-reinforced biocomposites, very few have addressed lifecycle assessments (LCAs), end-of-life degradation behaviour in diverse environmental settings (e.g., compost, soil, marine), or scalability of production using South African biomass feedstocks. Secondly, the performance of CNFs in ternary polymer blend systems (e.g., PLA/PCL/CNF or PLA/PBS/CNF) is still underexplored, especially concerning phase morphology, interfacial compatibilisation, and long-term performance under real-world application conditions.

Furthermore, there is a lack of standardisation in CNF extraction protocols, surface modification strategies, and composite formulation methods specific to maize husk-derived fibres. Without harmonised metrics, it remains challenging to scale and compare results across research efforts. South Africa’s limited biopolymer manufacturing infrastructure, regulatory uncertainty, and low public awareness present additional constraints on industrial translation.

## Figures and Tables

**Table 1:** Chemical composition of common agricultural residues and wastes [15,19].

Type of Biomass	Lignocellulosic Substrate	Cellulose (%)	Hemicellulose (%)	Lignin (%)
Agricultural waste	Corncob	45	35	15
	Wheat straw	36	30	19
	Sugarcane	38	23	22
	Rice straw	39	28	20
	Corn stover	33	31	11
	Bean straw	31	24	10
	**Maize husk**	**35**	**36**	**12**

## Abbreviations

The following abbreviations are used in this manuscript:

CNW	Cellulose nano-whiskers
EPR	Extended Producer Responsibility
LCA	Lifecycle assessment
PBS	Polybutylene succinate
PCL	Polycaprolactone
PLA	Polylactic acid
UV	Ultraviolet
